# *Fusarium ershadii* sp. nov., a Pathogen on *Asparagus officinalis* and *Musa acuminata*

**DOI:** 10.1007/s10658-017-1403-6

**Published:** 2018-01-15

**Authors:** Moslem Papizadeh, Anne D. van Diepeningen, Hamid Reza Zamanizadeh, Farkhondeh Saba, Hossein Ramezani

**Affiliations:** 1 Microorganisms Bank, Iranian Biological Resource Center (IBRC), Academic Center for Education, Culture and Research (ACECR), Tehran, Iran; 2 CBS-KNAW Fungal Biodiversity Centre, Uppsalalaan 8, 3584CT, Utrecht, The Netherlands; 3 BU Biointeractions and Plant Health, Wageningen University and Research, Droevendaalsesteeg 1, 6708PB, Wageningen, The Netherlands; 4 Department of Plant Pathology, College of Agriculture, Science and Research Branch, Islamic Azad University, Tehran, Iran; 5 Plants Bank, Iranian Biological Resource Center (IBRC), Academic Center for Education, Culture and Research (ACECR), Tehran, Iran

**Keywords:** *Fusarium solani species complex*, *Asparagus officinalis* pathogen, *Musa acuminata* pathogen

## Abstract

Two *Fusarium* strains, isolated from *Asparagus* in Italy and *Musa* in Vietnam respectively, proved to be members of an undescribed clade within the *Fusarium solani* species complex based on phylogenetic species recognition on ITS, partial RPB2 and EF-1α gene fragments. Macro- and micro-morphological investigations followed with physiological studies done on this new species: *Fusarium ershadii sp. nov* can be distinguished by its conidial morphology. Both isolates of *Fusarium ershadii* were shown to be pathogenic to the monocot *Asparagus officinalis* when inoculated on roots and induced hollow root symptoms within two weeks in *Asparagus officinalis* seedlings. In comparison mild disease symptoms were observed by the same strains on *Musa acuminata* seedlings.

## Introduction

Fusiform or banana-shaped multicelled conidia are probably the best known characteristic of the large Ascomycete genus *Fusarium*. Within the genus we find many plant pathogens, saprobes, mycotoxin producers, and an increasing number of human pathogens (*e.g.* Salah et al. 2015; van Diepeningen et al. 2014). For a long time, sections were recognized within the genus based on morphological characters. Nowadays, subdivisions within *Fusarium* are made in species complexes consisting of sibling species with limited to no morphological variation, which can be best discriminated based on sequence data. A plea has been made to keep most of the *Fusarium* species complexes of these agriculturally and for human health important species under the well-known genus denominator *Fusarium* rather than splitting the genus in nine or more different genera (Geiser et al. 2013).

One of the more basal clades within the genus *Fusarium* according to Geiser et al. (2013) is the *Fusarium solani* species complex (FSSC), centered on recently epitypified *Fusarium solani* (Schroers et al. 2016). However, Lombard et al. (2015) revisited various genera of *Nectriaceae* and suggested renaming the *Fusarium solani* species complex as *Neocosmospora* (Lombard et al. 2015), but we prefer to use Geiser’s proposal for a large *Fusarium* genus including virtually all *Fusarium* species of importance in plant pathology, medical mycology, mycotoxicology and basic research, and thus better recognized (Geiser et al. 2013). Members of FSSC are capable of causing disease on many agricultural important crops – often foot and root rots - and are the most commonly observed etiological agents of human fusarioses (Coleman 2015; O'Donnell et al. 2008).

Within the plant pathogenic fusaria it is common to talk about *formae speciales* describing the host plant species of an isolate. Host-specific virulence factors that determine the host or host range are usually located on dispensable supernumerary chromosomes. Within the *Fusarium oxysporum* species complex, host specificity and these supernumerary chromosomes were found to have been horizontally exchanged between different lineages and species (Baayen et al. 2000; Ma et al. 2013). However, *formae speciales* in FSSC seem to correspond to biologically and phylogenetically distinct species (Coleman 2015).

Based on multi-locus sequence analyses of core genome genes and regions, dozens of different phylogenetic lineages within the FSSC can be recognized (O'Donnell et al. 2008; Short et al. 2013; Zhang et al. 2006). Slowly, more of these lineages are described with Latin binomials and/or with more data regarding their ecological niches are published. Examples are the recent description of *Fusarium petroliphilum* (FSSC clade-1) and *F. keratoplasticum* (FSSC clade −2) (Short et al. 2013) and the epitypification of the potato dry rot pathogen *F. solani sensu stricto* (FSSC-clade-5) (Schroers et al. 2016). *Fusarium keratoplasticum*, a common inhabitant of soil, drainage systems and other antropogenic substrates, was recently suggested to be recombining, potentially even via heterothallic sex (Short et al. 2013; Short and O'Donnell 2014). A remarkable finding as *‘Fusarium solani´* is generally considered homothallic or asexual (O'Donnell et al. 2008).

In this paper we describe a new species within FSSC that also forms a monophyletic clade based on multiple loci. The strains were isolated from the monocots asparagus and banana and proved especially pathogenic on the first host. The new species was characterized morphologically and phylogenetically.

## Materials and methods

### Macroscopic and microscopic morphology

Morphological characteristics and growth rates were studied on potato dextrose agar (PDA), synthetic nutrient agar (SNA), and carnation leaf agar (CLA). Digital images of the colonies were documented and studied after 7 days of incubation at 25 °C, with and without UV (longer incubation up to a month was considered if needed). Inoculations were performed using a dense inoculum stock which was prepared from a 10-day-old colony on CLA medium. Macroscopic properties were studied on 10-day-old colonies. Slide cultures were mounted in a droplet of lactic acid or water to be studied with an Olympus BX51 microscope equipped with a DP25 digital imaging camera. Size of various structures was determined by averaging the measurements of 25–30 samples of each structure (Short et al. 2013).

### Growth rates

Single conidia of isolates, grown on Carnation Leaf Agar (CLA) (Fisher et al. 1982), were transferred to the center of 8.5 cm Potato Dextrose Agar (PDA) and Oatmeal agar (OA) plates and incubated in growth chambers at 25 °C and 30 °C. After 72 h, colony diameters were measured using a ruler and the average growth rate per isolates was calculated and expressed as colony growth rate per 24 h. Additionally, cardinal growth temperatures were determined on PDA plates that were mid-point inoculated and incubated at 5, 10, 15, 20, 25, 28, 30, 34, 37 and 40 °C for 7 days and any hyphal growth was studied under the light microscope (objective lenses 4X and 10X). The colony diameter was measured after 7 days (Hujslová *et al.* 2013; Selbmann *et al.* 2008).

Also, PDA and MEA media of different acidity (pH 3–8.5) were prepared in duplicate using a 2 M stock solution of HCl or NaOH (Hujslová *et al.* 2013; Selbmann *et al.* 2008). Plates were inoculated (single-point) and incubated at 28 °C for 10 days and diameter of the colonies was measured (Hujslová *et al.* 2013; Selbmann *et al.* 2008).

### Primers for molecular identification and phylogenetic analysis

Primers ITS1 (TCCGTAGGTGAACCTGCGG) and ITS4 (TCCTCCGCTTATTGATATGC) were used to amplify the ITS fragment (approximately 600 bp), primers EF1 (ATGGGTAAGGARGACAAGAC) and EF2 (GGARGTACCAGTSATCATGTT) were used to amplify a nearly 720 bp fragment of the coding gene for *EF-1α*, primers RPB2-5F (GAYGAYMGWGATCAYTTYGG) and RPB2-7R (CCCATWGCYTGCTTMCCCAT) were used to amplify a nearly 1200 bp fragment of the coding gene for *RPB2* (Geiser et al. 2013,; O'Donnell et al. 2007, O'Donnell et al. 2008.; Short et al. 2011; Zhang et al. 2006).

### DNA extraction and polymerase chain reaction

DNA was extracted using a manual purification procedure as described (Papizadeh et al. 2017a,; Saba et al. 2016). All the PCR amplifications were performed in a MyCycler™ thermal cycler system (BIORAD, USA). The 50 μl PCR mixtures were prepared with 1 μl DNA suspension, 5 μl of PCR buffer (Fermentas), 10 mmol of dNTPs, 2.5 mM MgSO4, and 10 pmole of each of the primers, 5 U of *PFU* DNA polymerase, 0.5 μl of absolute DMSO, and appropriate volume of DDW. A hot-start procedure (3 min, 94 °C) was used before the enzyme addition to prevent nonspecific annealing of the primers. All the PCR reactions for amplification of the ITS, and EF-1α fragments entailed 35 cycles (94 °C for 45 s, 56 °C for ITS [50 °C for EF-1α] for 50 s, 72 °C for 95 s, plus one additional cycle with a final 7 min chain elongation). For amplification of the selected fragment of the RPB2 gene the PCR conditions included: (1) hot start with 95 °C for 5 min; (2) 30 cycles of 1 min at 95 °C, 2 min at 55 °C (or 50 °C), an increase of 1 °C/5 s to 72 °C, and 2 min at 72 °C; and (3) a 10-min incubation at 72 °C, respectively. The PCR products were sequenced by Genfanavaran Biotech Corporation (O’Donnell et al. 2007). The DNA sequences determined for this study were submitted to GenBank, and the accession numbers for strain CBS 115.40 = IBRC-M 30232 are: KX503270 (RPB2), KX503269 (EF-1α), KX503267 (ITS). The accession numbers for strain CBS 139505 = IBRC-M 30096 KX503268 (ITS).

### Sequence analysis

Each of the DNA fragments was sequenced on both directions using the same primers which were used for PCR amplification. Sequences were assembled and edited with a trial version of Geneious software (www.geneious.com). Using the MEGA v. 7.0.9 package, sequences were aligned with sequences obtained from the online databases of CBS, NBRC, and GenBank (http://www.ncbi.nlm.nih.gov/). According to the results gained from the similarity assessments (CBS, *Fusarium* MLST, and NCBI), sequences were aligned with the multiple sequence alignment tool; Multiple sequence Alignment using Fast Fourier Transform (MAFFT), available at the European Bioinformatics Institute (EMBL-EBI) (Katoh et al. 2009, McWilliam et al. 2013). Alignments were manually improved in MEGA v. 7.0.9 and Bioedit v. 7.0.5.3 packages (default settings) (Tamura et al. 2011; Kumar et al. 2016). The flanking regions were excluded from the analysis. The alignments were checked visually and finally the resulting multiple sequence alignments were used for phylogenetic assessments. Concatenated multi-locus sequence alignments were prepared with the BioEdit 7.0.5.3 package. Phylogenetic trees were rooted with *Fusarium staphyleae* strain NRRL 22316. Phylogenetic analyses were performed for each dataset as well as with combined alignments consisting of ITS, EF-1α, and RPB2 regions.

The online tool Findmodel (http://www.hiv.lanl.gov/content/sequence/findmodel/findmodel. html) was used to determine the best nucleotide substitution model. Maximum likelihood (ML) distance analysis was conducted with the MEGA v. 7.0.9 package (Tamura et al. 2011) with the GTR + GAMMA substitution models. The robustness of the trees was evaluated by 1000 bootstrap replications. Bayesian analyses were conducted with MrBayes v3.2.1 (Huelsenbeck and Ronqvist 2001) executed on XSEDE (Extreme Science and Engineering Discovery Environment) through the CIPRES Science Gateway v3.3 (Miller et al. 2010) in two parallel runs, using the default settings but with these adjustments: general time reversible (GTR) model of DNA substitution as the best fit and a gamma distribution rate variation across sites (Huelsenbeck and Ronqvist 2001). This model was chosen as the result from a pretest with MrModeltest 2.2 (Nylander 2004). After this was determined, the GTR + I + G model, as the best nucleotide substitution model, was used for the combined ITS, EF-1α, and RPB2 dataset, and a MCMC heated chain was set with a temperature value of 0.05. The number of chains, number of generations, and sample frequencies were set respectively at 4, 50,000,000, and 1000. Chain convergence was determined using Tracer v1.5 (http://tree.bio.ed.ac.uk/software/tracer/) to confirm sufficiently large ESS values (>200). The sampled trees were subsequently summarized after omitting the first 25% of trees as burn-in using the “sump” and “sumt” commands implemented in MrBayes (Rambaut and Drummond 2009). Trees were visualized and edited using FigTree v1.4.2 (Rambaut 2008). The concatenated aligned dataset for ITS, EF-1α and RPB2 used in the analysis has been submitted with the TreeBASE under the submission ID 21561 (Papizadeh et al. 2017b).

### Phytopathogenicity tests

Strains of *Fusarium ershadii* (CBS 115.40 and CBS 139505) were grown on PDA for a week, the surface of the medium was removed using a sterile scalpel, and the mycelial material was added to 25 ml of sterile 0.05% tween80 solution. The suspension was vortexed for 20 min and then filtered through sterile cotton cloth to remove hyphae. Thereafter, conidia were counted using a haemocytometer and a suspension with an approximate density of 1.2 × 10^6^/ml was prepared. Roots of 11 months old *Asparagus officinalis* (Accession IBRC P1006759 of the Iranian Biological Reference Centre) seedlings, grown in greenhouse (25 °C and 12-h photoperiod), were washed in sterile water. Then, the roots of different seedlings were inoculated by immersion for a minute in the suspension of the conidia of strains CBS 115.40 and IBRC-M 30096, respectively. All tests were done in triplicate. Triplicate un-inoculated seedlings were used as control. Finally, all the seedlings were potted in sterilized-soil. Pots were incubated in a quarantined space in the greenhouse for three weeks and were examined and photographed in 3-day intervals. The same procedure was performed on two months old *Musa acuminata* (IBRC P1011416) seedlings produced by tissue culture.

## Results

### Phenotypic characterization

Strain CBS 115.40 was isolated by Bugnicourt in 1936 from *Musa sapientum* in Tonkin, Vietnam and was deposited in the CBS collection in 1940. The strain used to be the type strain of *Cylindrocarpon tonkinense*, a relative of *Cylindrocarpon lichenicola*. In 2002, Summerbell and Schroers showed that *C. lichenicola* falls within the FSSC, while it was noted that CBS 115.40 was a clearly distinct species also within the same *Fusarium solani* species complex (Summerbell and Schroers 2002). More recently CBS 139505 was isolated from diseased *Aspagarus* in Italy. Both strains proved to match in morphology and in multi-locus sequence analyses and we describe them here as *Fusarium ershadii*.

Morphological features of *F. ershadii* are shown in Table 1 and Fig. 2. Strains of *F. ershadii*, like other members of FSSC have septate, filiform conidiophores incorporating microconidia-bearing terminal monophialides. Although true macroconidia, characteristic of FSSC members, were not detected in dark nor under UV, some conidia, around 20 μm in length and 3-septate, were observed which may be assumed to be macroconidia (*e.g.* Fig 2 g and o). Chlamydospores were formed (*e.g.* Fig 2 s and t), sometimes directly from the mostly 1-septate conidia.

**Table 1:** Growth profile of *Fusarium ershadii*

pH	Colony Diam. (mm)	Temp.	Colony Diam. (mm)
3	No growth	5	No growth
3.5	12	10	7
4	29	15	28
5	46	20	39
5.5	56	25	57
6	58	28	59
7	57	30	59
7.2	56	34	22
8	52	37	19
8.5	43	40	No growth

Strains (CBS 115.40 and CBS 139505) showed the same macro- and micromorphology on agar plates with little to no pigmentation on the used media. Morphological characters are summarized in the species descriptions (Fig. 2). No growth was detected on MEA medium at 5 °C. Hence, 10 °C was recorded as the lowest temperature that the strains could grow. The strains did not grow at 40 °C and after a period at temperatures of 42 °C or higher they lost viability and were unable to grow at growth-permitting temperatures. The optimum temperature for growth was between 28 and 30 °C. The optimum pH value for growth was 6 (Table 1).

### Molecular Identification and phylogenetic analysis

**Fig. 1:**
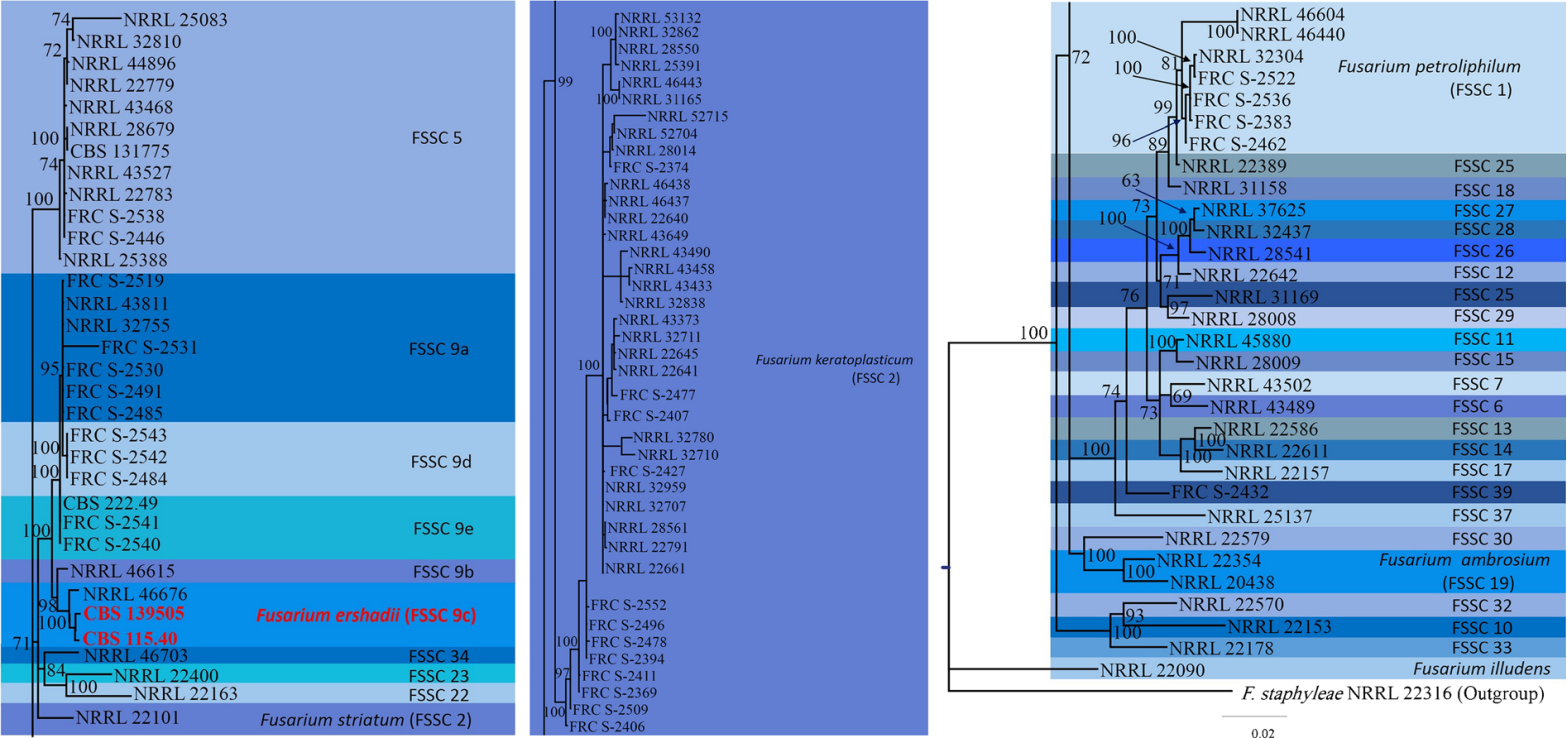
Phylogenetic relationships with maximum likelihood and Bayesian inference methods under the GTR + I + G model of evolution between Fusarium ershadii and the other members of FSSC based on the concatenated data of EF1-α, ITS and RPB2 (ML tree shown). At the branch tips the strain identifiers are given. In blocks species with latin binomials are indicated, but the majority of clades within FSSC do not have them yet. Clade 9c; *Fusarium ershadii*, Clade 5; *Fusarium solani senso stricto*, Clade 2; *F. keratoplasticum*, Clade 1; *F. Petroliphylum*. As outgroup *Fusarium staphyleae* NRRL 22316 was used

Sequences of three loci, EF-1α, RPB2, and ITS fragments, of *Fusarium ershadii* were studied in combination with sequences of FSSC isolates already available in the Fusarium MLST and GenBank databases (Fig. 1). The sequence identity in EF-1α fragments of 110 strains belonging to FSSC was about 56% (pairwise identity ~95%). For the ITS fragment sequence identity was around 64–68% (pairwise identity ~96.5%) and for RPB2 fragment 70–71% (pairwise identity ~97.5%).

Phylogenies performed on the combined set of ITS, EF-1α and RPB2 fragments and individual fragments resulted similar tree topologies. The analyses placed *F. ershadii* into a distinct clade (MLST group FSSC 9c) (Fig. 1). As is shown in Fig. 1, the posterior probability support for the *F. ershadii* clade was 0.9992/97% ML bootstrap value. The remainder of the tree was similar to that described for the FSSC (O’Donnell et al. 1998) (Table 2).

**Table 2:** Strains and sequences used in this study

Species complexes of Fusarium	Strain number	Sequence Accession Numbers			Reference
		RPB2	ITS	EF1-α	
*Fusarium ershadii* (9c)	CBS 115.40	KX503270.1	KX503267.1	KX503269.1	This study
*Fusarium ershadii* (9c)	CBS 139505		KX503268.1		This study
*Fusarium ershadii* (9c)	NRRL 46676	GU250731.1	GU250669.1	GU250546.1	Balmas et al. (2010)
FSSC 9b	NRRL 46615	GU250728.1	GU250666.1	GU250543.1	Balmas et al. (2010)
FSSC 9d	FRC S-2484	JN235906.1	JN235291.1	JN235721.1	Short et al. (2011)
FSSC 9d	FRC S-2542	JN235907.1	JN235292.1	JN235722.1	Short et al. (2011)
FSSC 9d	FRC S-2543	JN235908.1	JN235293.1	JN235723.1	Short et al. (2011)
FSSC 9a	FRC S-2519	JN235911.1	JN235296.1	JN235726.1	Short et al. (2011)
FSSC 9a	FRC S-2485	JN235909.1	JN235294.1	JN235724.1	Short et al. (2011)
FSSC 9a	FRC S-2491	JN235912.1	JN235297.1	JN235727.1	Short et al. (2011)
FSSC 9a	FRC S-2530	JN235910.1	JN235295.1	JN235725.1	Short et al. (2011)
FSSC 9a	FRC S-2531	JN235913.1	JN235298.1	JN235728.1	Short et al. (2011)
FSSC 9a	NRRL 32755	HM347159.1	DQ094534.1	DQ247073.1	Zhang et al. (2006)
FSSC 9a	NRRL 43811	EF470092.1	EF453204.1	EF453053.1	O'Donnell et al. (2007)
FSSC 9e	CBS 222.49	JX435259.1	JX435209.1	JX435159.1	Debourgogne et al. (2012)
FSSC 9e	FRC S-2540	JN235914.1	JN235299.1	JN235729.1	Short et al. (2011)
FSSC 9e	FRC S-2541	JN235915.1	JN235300.1	JN235730.1	Short et al. (2011)
FSSC 5	FRC S-2446	JN235917.1	JN235302.1	JN235732.1	Short et al. (2011)
FSSC 5	FRC S-2538	JN235942.1	JN235327.1	JN235757.1	Short et al. (2011)
FSSC 5	CBS 131775	JX237778.1	JX162380.1	JX118990.1	Zhang et al. (2006)
FSSC 5	NRRL 28679	EU329556.1	DQ094385.1	DQ246912.1	Zhang et al. (2006)
FSSC 5	NRRL 43468	EF469980.1	EF453093.1	EF452941.1	O'Donnell et al. (2007)
FSSC 5	NRRL 22779	EU329526.1	DQ094333.1	DQ246848.1	Zhang et al. (2006)
FSSC 5	NRRL 32810	EU329624.1	DQ094577.1	DQ247118.1	Zhang et al. (2006)
FSSC 5	NRRL 25083	JF740882.1	JF741044.1	JF740714.1	O'Donnell et al. (2012)
FSSC 5	NRRL 44896	GU170584.1	GU170639.1	GU170619.1	Migheli et al. (2010)
FSSC 5	NRRL 22783	EU329529.1	DQ094335.1	DQ246851.1	O'Donnell et al. (2007)
FSSC 5	NRRL 43527	EF470003.1	EF453116.1	EF452964.1	O'Donnell et al. (2007)
FSSC 5	NRRL 25388	EU329535.1	DQ094341.1	DQ246858.1	Zhang et al. (2006)
FSSC 22	NRRL 22163	EU329496.1	AF178394.1	AF178328.1	O'Donnell et al. (2008)
FSSC 23	NRRL 22400	EU329509.1	DQ094303.1	AF178343.1	O'Donnell et al. (2008)
FSSC 34	NRRL 46703	EU329661.1	EU329712.1	HM347126.1	O'Donnell et al. (2010)
*F. keratoplasticum* (FSSC 2)	FRC S-2427	JN235885.1	JN235270.1	JN235700.1	Short et al. (2011)
*F. keratoplasticum* (FSSC 2)	FRC S-2374	JN235767.1	JN235152.1	JN235582.1	Short et al. (2011)
*F. keratoplasticum* (FSSC 2)	NRRL 28014	EF470139.1	DQ094354.1	DQ246872.1	Zhang et al. (2006)
*F. keratoplasticum* (FSSC 2)	NRRL 43649	EU329639.1	EF453132.1	EF452980.1	O'Donnell et al. (2007)
*F. keratoplasticum* (FSSC 2)	FRC S-2407	JN235898.1	JN235283.1	JN235713.1	Short et al. (2011)
*F. keratoplasticum* (FSSC 2)	FRC S-2477	JN235897.1	JN235282.1	JN235712.1	Short et al. (2011)
*F. keratoplasticum* (FSSC 2)	NRRL 22641	EU329521.1	DQ094328.1	DQ246843.1	Zhang et al. (2006)
*F. keratoplasticum* (FSSC 2)	NRRL 22645	EU329523.1	DQ094330.1	DQ246845.1	Zhang et al. (2006)
*F. keratoplasticum* (FSSC 2)	NRRL 52715	JF741123.1	JF740912.1	JF740797.1	O'Donnell et al. (2012)
*F. keratoplasticum* (FSSC 2)	NRRL 32711	EU329597.1	DQ094493.1	DQ247031.1	Zhang et al. (2006)
*F. keratoplasticum* (FSSC 2)	NRRL 43373	EF469959.1	EF453072.1	EF452920.1	O'Donnell et al. (2007)
*F. keratoplasticum* (FSSC 2)	NRRL 32780	EU329617.1	DQ094551.1	DQ247090.1	Zhang et al. (2006)
*F. keratoplasticum* (FSSC 2)	NRRL 32959	EU329634.1	DQ094632.1	DQ247178.1	Zhang et al. (2006)
*F. keratoplasticum* (FSSC 2)	NRRL 22640	EU329520.1	DQ094327.1	DQ246842.1	Zhang et al. (2006)
*F. keratoplasticum* (FSSC 2)	NRRL 46437	GU170588.1	GU170643.1	GU170623.1	Migheli et al. (2010)
*F. keratoplasticum* (FSSC 2)	NRRL 46438	GU170589.1	GU170644.1	GU170624.1	Migheli et al. (2010)
*F. keratoplasticum* (FSSC 2)	NRRL 22661	EU329524.1	DQ094331.1	DQ246846.1	Zhang et al. (2006)
*F. keratoplasticum* (FSSC 2)	NRRL 22791	EU329530.1	DQ094337.1	DQ246853.1	Zhang et al. (2006)
*F. keratoplasticum* (FSSC 2)	NRRL 28561	EU329552.1	DQ094375.1	DQ246902.1	Zhang et al. (2006)
*F. keratoplasticum* (FSSC 2)	NRRL 25391	EU329536.1	DQ094343.1	DQ246860.1	Zhang et al. (2006)
*F. keratoplasticum* (FSSC 2)	NRRL 28550	EU329547.1	DQ094365.1	DQ246891.1	Zhang et al. (2006)
*F. keratoplasticum* (FSSC 2)	NRRL 32862	EU329631.1	DQ094621.1	DQ247167.1	Zhang et al. (2006)
*F. keratoplasticum* (FSSC 2)	NRRL 53132	GU170598.1	GU170654.1	GU170634.1	Migheli et al. (2010)
*F. keratoplasticum* (FSSC 2)	NRRL 31165	EU329562.1	DQ094394.1	DQ246921.1	Zhang et al. (2006)
*F. keratoplasticum* (FSSC 2)	NRRL 46443	GU170591.1	GU170646.1	GU170626.1	Migheli et al. (2010)
*F. keratoplasticum* (FSSC 2)	NRRL 32707	EU329595.1	DQ094490.1	DQ247027.1	Zhang et al. (2006)
*F. keratoplasticum* (FSSC 2)	NRRL 32710	EU329596.1	DQ094492.1	DQ247030.1	Zhang et al. (2006)
*F. keratoplasticum* (FSSC 2)	NRRL 32838	EU329627.1	EU329681.1	DQ247144.1	Zhang et al. (2006)
*F. keratoplasticum* (FSSC 2)	NRRL 43433	DQ790561.1	DQ790517.1	DQ790473.1	Chang et al. (2006)
*F. keratoplasticum* (FSSC 2)	NRRL 43458	EF470172.1	EU329686.1	DQ790511.1	Chang et al. (2006)
*F. keratoplasticum* (FSSC 2)	NRRL 43490	DQ790573.1	DQ790529.1	DQ790485.1	Chang et al. (2006)
*F. keratoplasticum* (FSSC 2)	FRC S-2394	JN235887.1	JN235272.1	JN235702.1	Short et al. (2011)
*F. keratoplasticum* (FSSC 2)	FRC S-2478	JN235888.1	JN235273.1	JN235703.1	Short et al. (2011)
*F. keratoplasticum* (FSSC 2)	FRC S-2496	JN235891.1	JN235276.1	JN235706.1	Short et al. (2011)
*F. keratoplasticum* (FSSC 2)	FRC S-2552	JN235846.1	JN235231.1	JN235661.1	Short et al. (2011)
*F. keratoplasticum* (FSSC 2)	FRC S-2369	JN235758.1	JN235143.1	JN235573.1	Short et al. (2011)
*F. keratoplasticum* (FSSC 2)	FRC S-2411	JN235772.1	JN235157.1	JN235587.1	Short et al. (2011)
*F. keratoplasticum* (FSSC 2)	NRRL 52704	JF741112.1	JF740908.1	JF740786.1	O'Donnell et al. (2012)
*F. keratoplasticum* (FSSC 2)	FRC S-2509	JN235788.1	JN235173.1	JN235603.1	Short et al. (2011)
*F. keratoplasticum* (FSSC 2)	FRC S-2406	JN235789.1	JN235174.1	JN235604.1	Short et al. (2011)
*F. striatum* (FSSC 21)	NRRL 22101	EU329490.1	AF178398.1	AF178333.1	Chehri (2014)
*F. petroliphilum* (FSSC 1)	FRC S-2383	JN235858.1	JN235243.1	JN235673.1	Short et al. (2011)
*F. petroliphilum* (FSSC 1)	FRC S-2522	JN235921.1	JN235306.1	JN235736.1	Short et al. (2011)
*F. petroliphilum* (FSSC 1)	NRRL 32304	EU329568.1	DQ094402.1	DQ246932.1	Zhang et al. (2006)
*F. petroliphilum* (FSSC 1)	FRC S-2536	JN235937.1	JN235322.1	JN235752.1	Short et al. (2011)
*F. petroliphilum* (FSSC 1)	FRC S-2462	JN235938.1	JN235323.1	JN235753.1	Short et al. (2011)
*F. petroliphilum* (FSSC 1)	NRRL 46440	GU170590.1	GU170645.1	GU170625.1	Migheli et al. (2010)
*F. petroliphilum* (FSSC 1)	NRRL 46604	GU170594.1	GU170649.1	GU170629.1	Migheli et al. (2010)
FSSC 25	NRRL 22389	EU329506.1	DQ094314.1	AF178340.1	Chehri (2017)
FSSC 18	NRRL 31158		DQ094389.1	DQ246916.1	Zhang et al. (2006)
FSSC 37	NRRL 25137	JF741084.1	JF740899.1	JF740757.1	Sandoval-Denis et al. (2018)
FSSC 29	NRRL 28008	EF470135.1	DQ094350.1	DQ246868.1	Zhang et al. (2006)
FSSC 25	NRRL 31169	KR673999.1	DQ094396.1	DQ246923.1	Zhang et al. (2006)
FSSC 26	NRRL 28541	EU329542.1	EU329674.1	DQ246882.1	Zhang et al. (2006)
FSSC 28	NRRL 32437	EU329581.1	DQ094446.1	DQ246979.1	Zhang et al. (2006)
FSSC 27	NRRL 37625	EU329637.1	EU329684.1	FJ240353.1	O'Donnell et al. (2008)
FSSC 12	NRRL 22642	EU329522.1	DQ094329.1	DQ246844.1	Zhang et al. (2006)
FSSC 39	FRC S-2432	JN235941.1	JN235326.1	JN235756.1	Short et al. (2011)
FSSC 7	NRRL 43502	DQ790576.1	DQ790532.1	DQ790488.1	Chang et al. (2006)
FSSC 15	NRRL 28009	EF470136.1	DQ094351.1	DQ246869.1	Zhang et al. (2006)
FSSC 11	NRRL 45880	EU329640.1	EU329689.1	FJ240352.1	O'Donnell et al. (2008)
FSSC 6	NRRL 43489	DQ790572.1	DQ790528.1	DQ790484.1	Chang et al. (2006)
FSSC 14	NRRL 22611	DQ094326.1	EU329518.1	DQ246841.1	Zhang et al. (2006)
FSSC 13	NRRL 22586	EU329516.1	DQ094312.1	AF178353.1	O'Donnell et al. (2009)
FSSC 17	NRRL 22157	EU329493.1	DQ094306.1	AF178359.1	O'Donnell et al. (2009)
FSSC 10	NRRL 22153	EU329492.1	DQ094302.1	AF178346.1	O'Donnell et al. (2009)
FSSC 32	NRRL 22570	EU329513.1	AF178422.1	AF178360.1	O'Donnell et al. (2009)
FSSC 33	NRRL 22178	EU329498.1	DQ094313.1	AF178334.1	O'Donnell et al. (2009)
*F. ambrosium* (FSSC 19)	NRRL 20438	JX171584.1	DQ094315.1	AF178332.1	O'Donnell et al. (2009)
*F. ambrosium* (FSSC 19)	NRRL 22354	EU329504.1	DQ094316.1	AF178338.1	O'Donnell et al. (2009)
FSSC 30	NRRL 22579	EU329515.1	AF178415.1	AF178352.1	O'Donnell et al. (2009)
*F. illudens*	NRRL 22090	JX171601.1	AF178393.1	AF178326.1	O'Donnell et al. (2009)
*F. staphyleae*	NRRL 22316	JX171609.1	AF178423.1	AF178361.1	O'Donnell et al. (2009)

#### Phytopathogenicity

We tested our two strains for pathogenicity on the two host plants they were isolated from. The same pathologic results were seen on the triplicate inoculated plants of *Asparagus officinalis* and *Musa acuminata*. Our phytopathogenicity tests showed that the inoculation of roots of *Asparagus* plants (*Asparagus officinalis* IBRC P1006759) with strains CBS 139505 and CBS 115.40 led to a severely reduced growth within 10 days (Figure. 3). Furthermore, the roots showed clearly ‘hollow root’-like symptoms with strong pigmentation within root tissues and the formation of empty areas void of plant material. These symptoms are comparable to the symptoms of *Fusarium* crown and root rot in asparagus, normally attributed to *Fusarium oxysporum* f.sp. *asparagi*, *F. proliferatum*, unspecified *F. solani*, and *F. redolens* (Elmer 2015). In comparison, inoculation of roots of banana plants (*Musa acuminata* IBRC P1011416) with the same strains, resulted in a reduced growth but not to the level that was seen on *Asparagus* plants (Fig. 3d-e).

## Taxonomy

**Fig. 2:**
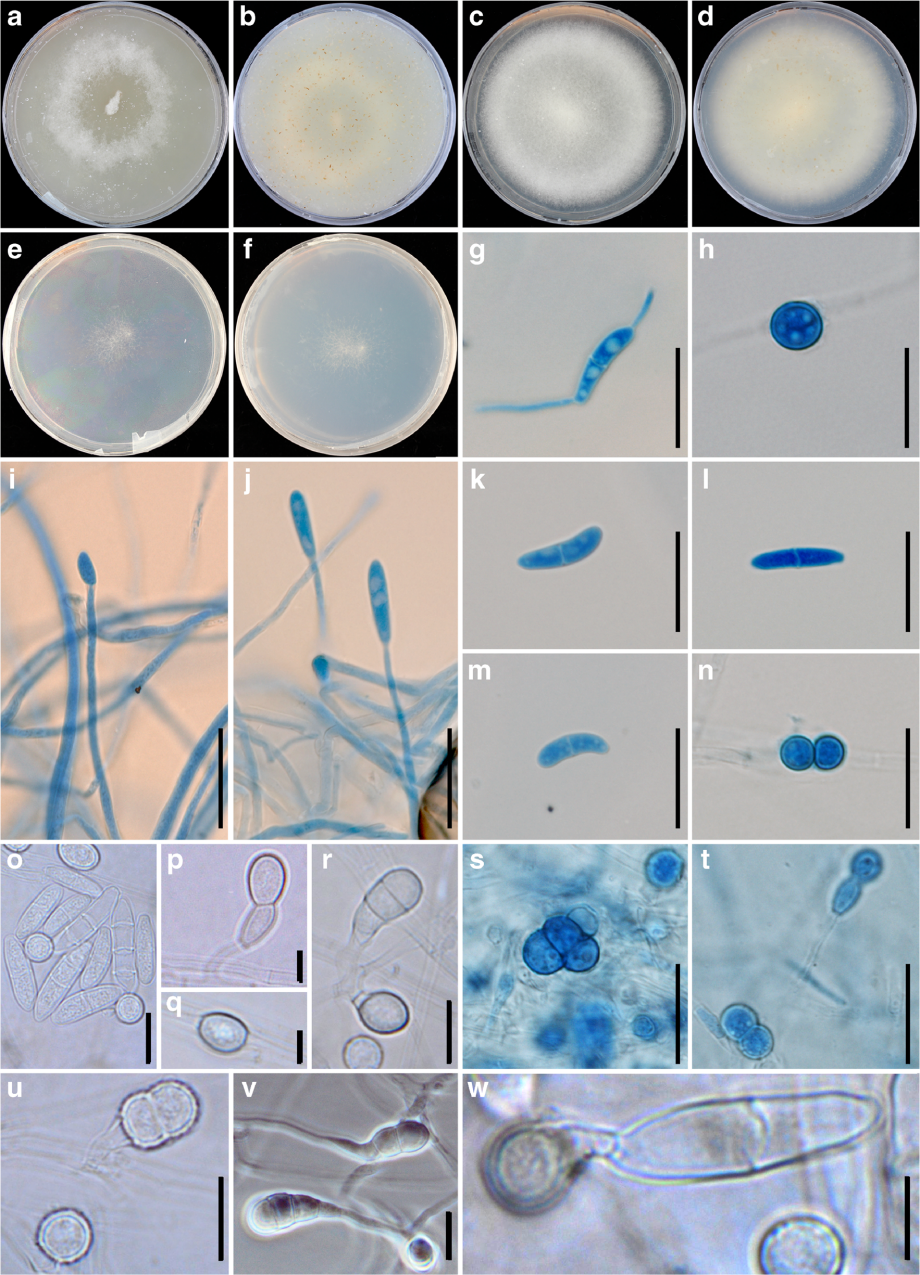
Morphological properties of Fusarium ershadii (strain CBS 115.40 and CBS 139505). a-b. Fast-growing 10-day old colony on oatmeal agar (OA) front and back side, growth rate approx. 0.45 cmday-1; c-d. Ten-day old colony on potato dextrose agar (PDA) front and back side, growth rate approx. 0.43 cmday-1; e-f. Ten-day old colony on synthetic nutrient agar (SNA) front and back side, growth rate approx. 0.43 cmday-1: the strain has covered the whole plate area with thin mycelium; g. Germinating microconidium (PDA 1000×, cotton-blue stained); h. Single chlamydospore; i-j. Monophialidic conidiophores of aerial mycelium; K-l. 1-septate oval Microconidia, (PDA 1000×, cotton-blue stained); m. 1-septate reniform microconidium; n. pairs of chlamydospores; o. 1–3 septate oval microconidia; p-w various forms of chlamydospores; x. Chlamydospore formed on microconidium. All scale bars 10 μm

*Fusarium ershadii* Papizadeh, van Diepeningen, & Zamanizadeh, *sp. nov.* (Figs. 1 and 2).

**Fig. 3:**
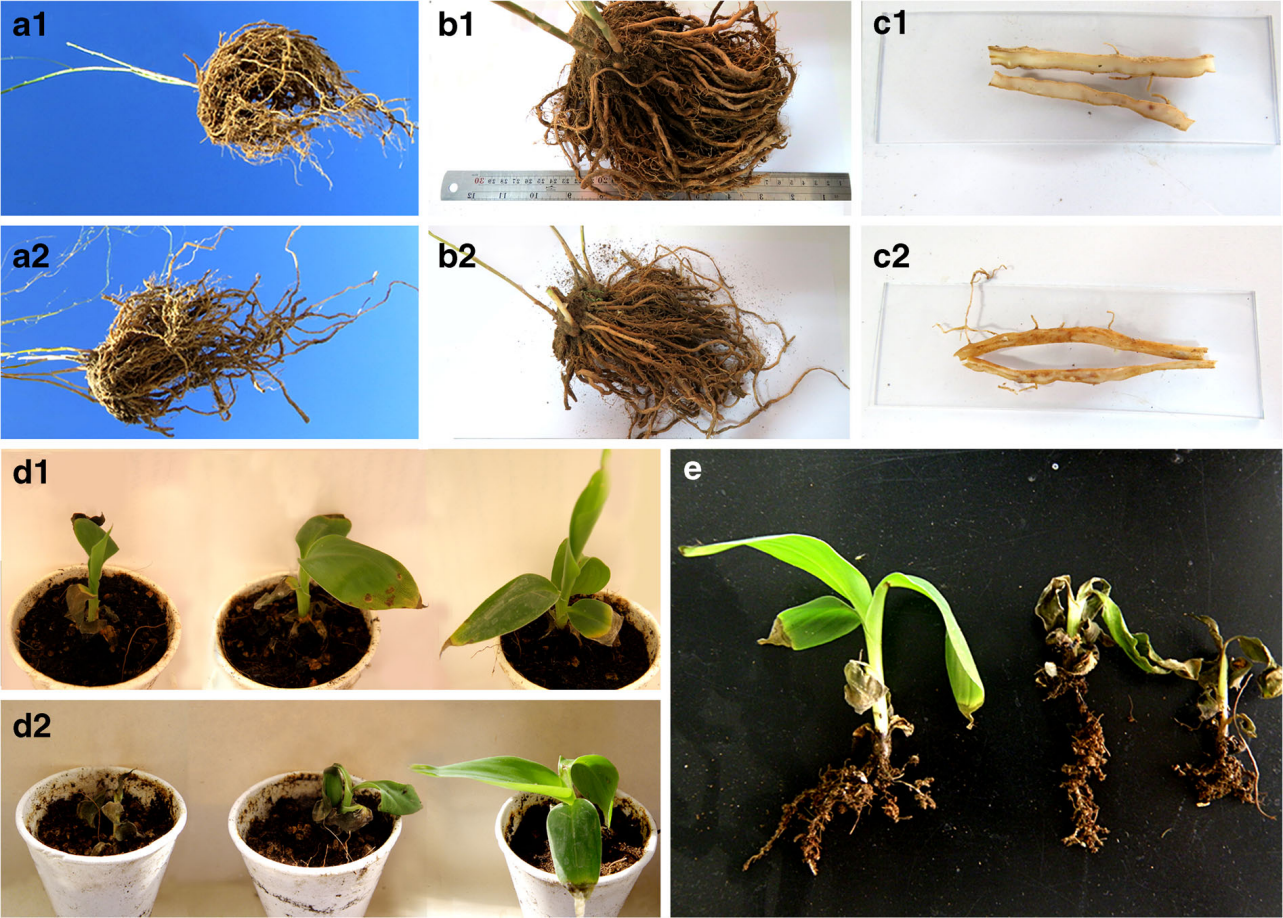
Phytopathogenicity of *Fusarium ershadii* strains on *Asparagus officinalis* (A, B, and C) and *Musa acuminata* (D and E). Hollow root disease symptoms in *Asparagus officinalis* induced by inoculation of *Fusarium ershadii* strains (un-inoculated; A1, B1, and C1, inoculated; A2, B2, and C2). Inoculation of the roots of the *Musa acuminata* with strains of *Fusarium ershadii*. The pathogenicity test after one (D1) and two weeks (D2), from right; un-inoculated, inoculated with CBS 139505, and inoculated with CBS 115.40. *Musa acuminata* seedlings, from left; un-inoculated, inoculated with CBS 139505, and inoculated with CBS 115.40

Mycobank: MB 817602.

Type: Vietnam, Tonkin, isolated from *Musa sapientum*, 1936, collected by F. Bugnicourt. (holotype: IBRC-H 2025, a dried culture) [Ex-type: CBS 115.40^T^ = IBRC-M 30232^T^ preserved in a metabolically inactive state (cryopreserved)].

Additional strain: CBS 139505 = IBRC-M 30096.

Sequences from ex-type culture, CBS 115.40: ITS (KX503267), EF-1α (KX503269), RPB2 (KX503270) and from strain CBS 139505 = IBRC-M 30096: ITS (KX503268).

Etymology. Species epithet *ershadii* is selected in honor of Prof. Djafar Ershad for his contribution to mycology in Iran.

Conidia 12–20 μm in length, thick-walled, 1–3 septate oval, predominantly 1-septate oval (k-m & o), with little distinction in size between micro and macroconidia. Reniform microconidia rarely detected (m). Conidiophores elongate (50–130 μm), filiform, 1–3 septate, incorporating microconidia-bearing terminal monophialides (i & j). Chlamydospores smooth-walled (p-s) or verrucose-verroculose (u), mostly intercalary, but also terminal. Singular intercalary chlamydospores globose to subglobose with or without supporting cells (4.5–7.5 μm in diam) (h, q & r). Pairs (n) and clusters of 2–4 celled globose to subglobose smooth-walled chlamydospores (4.5–7.5 μm in diam) with (p-w) or without supporting cells (s).

Culture characteristics- Colonies fast-growing, growth rate on oatmeal agar (OA), 0.45 cmday^−1^; a-b. Growth rate on potato dextrose agar (PDA), 0.43 cmday^−1^; c-d. Growth rate on synthetic nutrient agar (SNA) 0.43 cmday^−1^; e-f. The strain has covered the Petri dish with thin mycelium. Colonies attaining 29 mm diam. in 7 d on PDA at 20 °C, 57 mm diam. at 25 °C, 59 mm diam. at 28–30 °C, 22 mm diam. at 34 °C, and 19 mm at 37 °C. Fungus did not grow at 5 and 40 °C and it became unviable at 42 °C. Incubating under an alternating day/night 12 h photoperiod. The fungus grew at a broad range of pH (3.5–8.5), while the optimum growth occurred at pH values between 5.5 and 6.5.

## Discussion

*Fusarium ershadii* forms a well-supported monophyletic lineage within clade 9 of FSSC and can be distinguished from all other species in this group based on DNA sequence comparisons and morphology. *Fusarium ershadii* seems to occur at least in North-America, Europe and Asia as a saprobe and as a pathogen. Here we have shown that the new species is a strong pathogen on *Asparagus officinalis* and a weak pathogen on *Musa acuminata*.

Genealogical concordance phylogenetic species recognition (GCPSR) (Taylor et al. 2000) is based on the concordance of multiple gene genealogies. In this study three loci; EF-1α, RPB2, and ITS, were used for GCPSR analyses, singly and concatenated. The EF-1α and RPB2 fragments used have high levels of variation and are suitable barcodes for *Fusarium* (*e.g.* Al-Hatmi et al. 2016), while ITS is the general DNA barcode for fungi (Schoch et al. 2012), all three barcodes have the benefit that public repositories like Genbank contain large numbers of them for fungi of the genus *Fusarium*. Sequence analysis of the combined set of ITS, EF-1α and RPB2 showed similar results gained from each fragment individually. Besides, according to the sequence identity and average pairwise identity values gained from the sequence analysis of EF-1α, RPB2, and ITS fragments, it can be inferred that the ITS fragment has a significant resolution in FSSC and it is quite different from what is seen in other species complexes of *Fusarium* (Papizadeh et al. 2015 and Papizadeh et al. 2016). Such a different resolution power of ITS fragment in FSSC conforms to the fact that FSSC forms a basal clade in the genus *Fusarium* with a significant phylogenic distance from the other clades of the genus. Additionally, although EF-1α fragment, as the most variable fragment in FSSC, is recommended as the first choice for species delimitation in FSSC, the complementary effects of ITS and RPB-2 fragments should not be neglected, because they cause a higher robustness in phylogeny studies which can be inferred from the bootstrap and posterior probability values.

In general, the growth profile of *F. ershadii* seems to be similar to that of *F. keratoplasticum* (FSSC 2), *F. petroliphilum* (FSSC 1), and *F. solani* s.s. (FSSC 5) (Short et al. 2013; Schroers et al. 2016). The optimum temperature for growth of *Fusarium ershadii* is 28–30 °C, this temperature varies little in the described clades of FSSC (Short et al. 2013). Besides, growth rate studies on a pH gradient of showed that *Fusarium ershadii* can grow well in pH values between 3.5 and 8.5. However, the optimum pH was around 6 and a higher growth rate was recorded in mildly alkaline pH conditions (7.2–8.5) in comparison to mildly acidic pH values (4–5). While CBS 114.50 was first considered to be a close relative of *F. lichenicola* previously called *Cylindrocarpon lichenicola* (Summerbell and Schroers 2002)*, F. ershadii* proves to be closer to the type species of *F. solani* than to *F. lichenicola* (lineage 16).

Morphologically, forming a diverse range of chlamydospores is a character which can be assumed as one of the main characteristics of FSSC. *F. keratoplasticum* and *F. petroliphilum* have also been described with such chlamydospores (Short et al. 2013). No sporodichia were detected to compare the morphology of sporodochial conidia of *Fusarium ershadii* to the other members of FSSC. Interestingly, aerial conidia of *F. ershadii* were 12–20 μm in length which is shorter than those of *F. keratoplasticum*, *F. falciforme*, *F. petroliphilum* and *F. solani* s.s. However, morphology and dimension of these conidia are highly similar to those of *F. keratoplasticum*.

Members of FSSC are cosmopolitan soil-borne hyphomycetes. These fusaria are also detected as pathogens not only of humans and other animals, but also a diverse range of plants. Based on sequence analyses, several additional strains were identified in Genbank as members of *F. ershadii*. The origins of these strains indicate a worldwide spread as saprobes and/or pathogens, as they include soil isolates from Sardinia, Italy (Balmas et al. 2010), a corn root isolate from Illionois, USA (Zhang et al. 2006), and a Chinese isolate from sugar beet (Cao and Wu, unpublished results). *Fusarium ershadii* also has a special niche causing hollow root disease in *Asparagus* plants. It may have potential as a pathogen of other monocots such as banana and maize. *Formae speciales*, specialized on certain host crops in FSSC, are assumed to correspond to biologically and phylogenetically distinct species (Coleman 2015), whereas within the *Fusarium oxysporum* species complex host specificity encoded on supernumerary chromosomes were found to have been horizontally exchanged between different lineages and species (Baayen et al. 2000; Ma et al. 2013).

The *Fusarium solani* species complex is one of the more basal clades within the genus *Fusarium* according to the concept of Geiser et al. (2013); or, following Lombard et al. (2015) the clade would be called *Neocosmospora* (Lombard et al. 2015). The FSSC contain several members causing root rots and hollow roots and as *Fusarium* is the better known genus, we adhere to the first concept of a large monophyletic genus *Fusarium* and adhere to *Fusarium ershadii* for this new *Asparagus* pathogen.
